# The SWAT (study within a trial) programme; embedding trials to improve the methodological design and conduct of future research

**DOI:** 10.1186/1745-6215-16-S2-P209

**Published:** 2015-11-16

**Authors:** Mike Clarke, Gerard Savage, Lisa Maguire, Helen McAneney

**Affiliations:** 1 Queen's University Belfast, Belfast, UK; 2 University of Liverpool, Liverpool, UK

## 

Researchers and trialists face many uncertainties when designing and conducting research. Embedded methodology studies can help to resolve these. However, despite hundreds of thousands of trials, there are probably only a few hundred studies assessing the effects of different methods for doing this research. The concept of the SWAT (Study Within A Trial) programme is to aid the development of such research by increasing awareness of, and stimulating interest in the need for this research and providing a framework and resource to inspire and generate ideas, and to store, disseminate and modify such research. The programme was established as part of the development of an All-Ireland Hub for Trials Methodology Research in collaboration with the Medical Research Council's Network of Hubs in the UK and the Global Health Network. It will facilitate this research into research. Each SWAT comprises of simple, one or two page protocols and, as of April 2015, 19 different SWAT outlines have been registered. This presentation will detail the SWAT concept, and describe the core outline which consists of the following sections: background, intervention, comparator, allocation, primary outcomes, secondary outcomes, analysis, possible problems, likely costs, publications, and version information. The SWAT website will be available to view, and examples of completed SWATs will be shown. The presentation should stimulate ideas for future SWAT, and encourage researchers to see how they might maximize the impact of embedding research into research, leading to improvements in the design of future clinical trials and other studies.

